# Nutritional quality and *in vitro* digestibility of fermented rice bran based on different types and doses of inoculants

**DOI:** 10.5455/javar.2022.i632

**Published:** 2022-12-31

**Authors:** Syamsul Hidayat Dilaga, Ryan Aryadin Putra, Anggriawan Naidilah Tetra Pratama, Oscar Yanuarianto, Muhamad Amin, Suhubdy Suhubdy

**Affiliations:** 1 Laboratory of Ruminant/Herbivore Nutrition, Faculty of Animal Science, University of Mataram, Mataram, Indonesia; 2 Department of Animal Science, Faculty of Agriculture, Sriwijaya University, Palembang, Indonesia

**Keywords:** Bran, *Saccharomyces cerevisiae*, effective microorganism, feed burger sauce

## Abstract

**Objective:**

The study was conducted to determine the effect of inoculants of different types and doses on the nutrient quality and *in vitro* digestibility of fermented rice bran.

**Materials and Methods:**

The study was designed using a completely randomized design with a 3 × 3-factorial pattern. The first factor was the type of inoculum, consisting of *Saccharomyces cerevisiae* (SC), Effective Microorganism-4, and Saus Burger Pakan (SBP). The second factor is inoculum doses, which are as follows: levels 2%, 4%, and 6%. The variables measured included chemical composition, fiber fraction content, dry matter digestibility and organic matter digestibility.

**Results:**

The results showed that the type of inoculation treatment and the doses of inoculation did not affect the dry matter (DM) content of fermented bran, and the organic matter content of fermented bran was only affected by the inoculation dose factor (*p *< 0.05). The highest crude protein and Extract Ether (EE) were obtained in the SBP inoculants, which increased linearly with increasing inoculation doses (*p *< 0.05). While a significant decrease (*p *< 0.05) occurred in crude fiber content. The cellulose, hemicellulose, lignin, acid detergent fiber (ADF), and neutral detergent fiber (NDF) fractions were significantly lower in the SBP treatment as the dose increased. The SBP inoculant type produced the highest DMD (*p *< 0.05) but showed a response that was not different from the SC inoculant treatment for OMD. Increasing inoculation doses of 2%, 4%, and 6% linearly increased the DMD and OMD of fermented bran (*p *< 0.05). Overall, inoculant application on fermented bran showed an interaction effect except for the components of DM, EE, ADF, NDF, and DMD of fermented bran.

**Conclusions:**

It was concluded that the SBP at 6% and their combination resulted in the best chemical quality and digestibility of rice bran.

## Introduction

Rice bran is one of the agricultural by-products abundant in rice-based agricultural countries such as Indonesia and can potentially be a feed ingredient [1]. The bran is obtained as the main by-product of the process of exfoliating the husks of unhulled rice and grinding broken rice [2]. Produced in large quantities worldwide, utilized as cheap feed for cattle and poultry [3], and contains important nutrients and bioactive compounds related to health [4]. Previous research that we have done shows that there is a very contrasting quality difference between the bran produced by a static huller (single-step huller) and the bran produced by a mobile huller (multi-pass huller). The cause of these differences is thought to be caused by differences in the workings of the milling machines used [5]. Thus, an effort to improve the quality of the bran is to utilize the services of microorganisms through the fermentation process.

The most recent sustainable strategy to maximize the utilization of bioresources in resolving the food supply crisis was fermentation [6]. The fermentation process and the use of specific enzymes have been extensively studied with the main aim of improving the overall characteristics of the raw material being processed [7]. The source of the inoculant has a major influence on the characteristics of the fermentation results. The difference in fermented product quality is largely determined by the different metabolic capabilities and specifications of the inoculum used as a fermenter agent. Fermentation can increase the nutritional quality of bran while decreasing anti-nutritional elements in the ingredients [8]. The purpose of this study was to determine whether several inoculants at different doses could produce the best-quality fermented bran with increased nutritional quality and digestibility.

## Materials and Methods

### Sampling and inoculant preparation

The research material in the form of rice bran used in this study was obtained from a rice mill located on the Lombok Island, Indonesia. The bran used as research material is taken randomly from East Lombok, Central Lombok, West Lombok, and North Lombok. After the bran collection process is complete, all the collected bran is mixed until it is homogeneous and then sampled for analysis of its chemical composition (Table 1). The fermentation inoculum in the form of *Saccharomyces cerevisiae* (SC) was obtained from commercial tempe yeast; effective microorganism-4 (EM4) was obtained from sales agents in Mataram City, and Saus Burger Pakan (SBP) was obtained from CV. Agromix Lestari Yogyakarta. Finally, the fermentation process is carried out on a laboratory scale using polyester plastic as a fermentation medium.

### Fermentation process and in vitro incubation

The inoculants were dissolved in distilled water and mixed with 500 gm of rice bran samples for each treatment. A fermented solution is then separated into 50 ml treatments with concentrations of 2%, 4%, and 6% of each type of inoculant.

After harvesting (14 days), 200 gm of fermented bran samples were sampled for the purposes of chemical composition analysis, such as dry matter (DM), organic matter (OM), crude protein (CP), crude fiber (CF), and Extract Ether (EE), determined based on the procedure [9]. Fiber fractions such as cellulose, hemicellulose, acid detergent fiber (ADF), neutral detergent fiber (NDF), and lignin were determined following the Van Soest procedure [10]. Meanwhile, for the purpose of *in vitro* digestibility testing, 0.5 gm of the sub-sample was weighed for testing on the level of digestibility. The digestibility values of DM and OM were determined based on the *in vitro *method by Tilley and Terry [11].

**Table 1.** Nutrient content of rice bran from mobile rice mills obtained from various locations on the Island of Lombok.

Chemical composition	Content percentage
DM	90.61
OM	83.49
CP	5.13
CF	29.73
EE	3.26

### Experimental design

In this study, the experimental design used was a completely randomized factorial pattern in which two factors were tested, namely the type and dose of inoculants. The treatments were as follows: SC with 2% inoculation dose; SC with 4% inoculation dose; SC with 6% inoculation dose; EM4 with 2% inoculation dose; EM4 with 4% inoculation dose; EM4 with 6% inoculation dose; SBP with 2% inoculation dose; SBP with 4% inoculation dose; and SBP with a 6% inoculation dose. All bran samples were fermented for 14 days.

### Data analysis

The data will be processed using Statistical Product and Service Solutions version 20 software, based on the design used. In addition, Duncan’s New Multiple Range Test will be tested to see if there are differences between treatments.

## Results

### Chemical composition

The results showed that the feeds value of DM content did not show significant results in all treatments (*p *> 0.05). However, different results were shown by the OM content. There was a significant difference between treatments in SC and SBP treatment at all doses compared to EM4 treatment (*p *< 0.05). In contrast, in EE content, a significant difference was shown in the SBP treatment with a 4%–6% dose. Fermented bran OM was significantly influenced by the type of inoculant and its interaction with the inoculation dose (*p* < 0.05). In contrast, the dose of inoculation treatment only partially affected the OM content of fermented bran. SC and SBP inoculation treatments had no different OM content. However, the SC and SBP treatments were significantly higher than the OM content of the EM4 inoculation treatment (83.76% *vs.* 85.25% and 85.37%; *p* < 0.05).

Observing the fiber and CP content values also revealed changes in the composition of nutrient content. However, the two variables had different patterns; CP showed the highest value in the SBP treatment at all doses (2%–6%) but was not significantly different compared with the EM4 treatment at a dose of 6%. While CF content was low, the highest value was found in the SBP treatment of 2%, which did not differ from 4%. The values obtained for SC and EM4 treatments at each dose showed an increasing trend with increasing inoculation doses.

Table 2 shows the effect of the type of inoculant, the dose of inoculation, and their interaction of the two treatments on the CP content of fermented bran (*p* < 0.05). The CP content of fermented bran with SBP was significantly higher than that of SC and EM4 treatments (6.92% *vs.* 5.77% and 5.77%, respectively; *p* < 0.05). In addition, the treatment of inoculation type and dose, as well as the interaction between type and dose of inoculation, significantly affected the CF content of fermented bran (*p* < 0.05). In percentage terms, the decreased CF content due to the effect of the type of inoculation ranged from 1.95% to 4.51%.

The data in Table 2 showed that the type and dose of inoculation had a significant effect (*p *< 0.05), but the interaction of both treatment factors did not show a significant response to the EE content of fermented bran. SC inoculants significantly produced lower EE than EM4 and SBP inoculations (2.98 *vs.* 3.89; 5.14; *p *< 0.05). EM4 and SBP inoculations also showed different responses, with lower EE produced by fermentation using EM4 than SBP (3.89 *vs*. 5.14; *p *< 0.05).

### Fiber fraction

The results showed that the value of cellulose and lignin expressed a significant change in SC treatment with a dose of 2% compared to other treatments. However, increasing the treatment dose for each type of inoculant showed a downward trend in the value of each variable. The cellulose content of fermented bran was significantly influenced by the type and dose of inoculants and their interactions (*p* < 0.05). The data in Table 2 indicate that the use of SBP resulted in the lowest cellulose content (17.42%) but did not show any difference with the cellulose content of the EM4 inoculant treatment (17.43%). The SC treatment produced high cellulose compared to the other treatments, which were 19.50% (*p *< 0.05). Likewise, the effect of the inoculant dose showed a linearly decreasing trend in line with the increasing doses. The inoculation dose of 6% resulted in the lowest cellulose content of 14.75%. While the treatment doses of 2% and 4% had cellulose contents of 21.22% and 18.56%, respectively (*p* < 0.05).

The same results were also shown in the ADF and NDF values, where the highest value was found in the 6% dose of EM4 treatment. Furthermore, in the observation of the hemicellulose content, significant changes occurred in the EM4 treatment with a dose of 2%–6% compared to other treatments but did not differ when compared to the SBP treatment of 2%–4%. The type of inoculant showed a significant effect (*p *< 0.05), but treatment doses did not significantly affect the hemicellulose content. A significant effect was shown by the interaction of the two treatment factors.

### Dry matter and organic matter digestibility

The results showed that DM and organic matter digestibility (OMD) significantly differed in SBP treatment at a 4%–6% dose. However, dry matter digestibility (DMD) showed no interaction, while OMD showed a strong interaction between treatment variables.

The DMD of fermented bran was significantly influenced by the type and dose of inoculum (*p *< 0.05), but the two treatment factors did not show any interaction effect. The application of SBP significantly resulted in the highest DMD (41.33%), followed by SC inoculation treatment (39.34%), and finally, EM4 treatment, which produced the lowest DMD (34.90) (*p* < 0.05).

The results of the DMD measurement of fermented bran were significantly influenced by the doses of inoculant (*p* < 0.05). The OMD value of fermented bran ranged from 36.91% to 40.18%. The OMD in the 6% treatment was higher than 2% and 4% inoculation treatments (40.18 *vs.* 36.91 and 38.48%, *p *< 0.05).

## Discussion

### Dry Matter and Organic Matter content

The results of the statistical analysis showed that there was no effect of the type of inoculum treatment, the inoculation dose, and their interactions on the DM content of fermented bran (Table 2). This result is the same as that in [12], which showed that SC inoculation did not affect the DM content of fermented bran. However, the results of research conducted on corn silage showed that adding SC alone or in a mixture resulted in changes in the chemical composition of feed ingredients [13]. Furthermore, other types of inoculants with different doses produce the same results, confirming the suspicion that providing inoculants during the fermentation process using high-carbohydrate substances will not result in changes in DM, especially because high carbohydrates are easily soluble in the feed ingredients, causing the substrate from fermentation that is formed to produce lactic acid, which lowers the pH in the fermentation process [14–17]. So it could be assumed that the role of existing inoculants is not so significant in maintaining feed nutrients as the role of dissolved carbohydrates, which have a real influence in maintaining feed nutrients. The results showed that fermentation using feed ingredients with a high energy content without the use of precursor bacteria (lactic acid bacteria) could create acidic conditions with a low pH during the fermentation process because lactic acid bacteria that are naturally present in the feed ingredients will appear due to the availability of easily dissolved carbohydrate content [17–19].

**Table 2.** Nutrient composition and digestibility of rice bran fermented with different types and doses of inoculant.

Variable	SC			EM-4			SBP			*SEM*	*p-*value		
	2%	4%	6%	2%	4%	6%	2%	4%	6%		Type	Doses	T x D
**Chemical composition, % DM**													
DM	82.85 ± 0.16	83.28 ± 0.18	83.32 ± 0.52	83.35 ± 0.36	83.14 ± 0.50	82.97 ± 1.19	83.23 ± 0.44	83.16 ± 0.88	82.68 ± 0.27	0.342	0.868	0.745	0.612
OM	85.21^c^ ± 0.10	84.97^c^ ± 0.18	85.57^c^ ± 0.04	84.01^b^ ± 0.21	84.13^b^ ± 0.04	83.15^a^ ± 0.72	85.19^c^ ± 0.11	85.45^c^ ± 0.24	85.47^c^ ± 0.51	0.187	<0.001	0.744	0.004
CP	5.53^a^ ± 0.30	5.88^bc^ ± 0.05	5.92^bc^ ± 0.02	5.52^a^ ± 0.05	5.67^ab^ ± 0.18	6.11^cd^ ± 0.18	6.26^d^ ± 0.05	6.27^d^ ± 0.02	6.32^d^ ± 0.23	0.090	<0.001	0.001	0.041
CF	26.34^c^ ± 0.54	25.24^b^ ± 0.23	24.08^a^ ± 0.22	26.07^c^ ± 0.03	25.83^bc^ ± 0.11	25.43^b^ ± 0.10	28.19^e^ ± 0.42	27.67^de^ ± 0.50	27.44^d^ ± 0.44	0.197	<0.001	<0.001	0.005
EE	2.61^a^ ± 0.24	3.13^b^ ± 0.35	3.20^b^ ± 0.16	3.46^b^ ± 0.02	4.00^c^ ± 0.05	4.20^c^ ± 0.09	4.66^d^ ± 0.12	5.25^e^ ± 0.14	5.51^e^ ± 0.39	0.119	<0.001	<0.001	0.872
GE (kcal/kg)	3,301 ± 7.85	3,163 ± 5.15	3,185 ± 2.39	3,269 ± 7.56	3,223 ± 3.51	3,145 ± 5.90	3,361 ± 5.52	3,185 ± 6.15	3,176 ± 8.05				
**Fiber fraction, % DM**													
Cellulose	22.43^g^ ± 0.39	20.63^e^ ± 0.34	15.44^b^ ± 0.18	19.80^d^ ± 0.43	17.37^c^ ± 0.21	13.47^b^ ± 0.13	21.44^f^ ± 0.43	17.37^c^ ± 0.21	13.47^a^ ± 0.13	0.162	<0.001	<0.001	<0.001
Hemicellulose	11.11^a^ ± 0.24	12.02^ab^ ± 1.46	13.42^bc^ ± 0.77	14.31^e^ ± 0.18	14.34^e^ ± 0.23	14.24^e^ ± 0.62	13.98^de^ ± 0.31	13.91^de^ ± 1.30	12.63^bcd^ ± 1.10	0.481	<0.001	0.695	0.017
Lignin	18.73^g^ ± 0.85	16.44^e^ ± 0.05	13.62^c^ ± 0.10	17.65^f^ ± 0.06	15.51^d^ ± 0.35	12.50^b^ ± 0.25	12.90^b^ ± 0.23	10.50^a^ ± 0.18	10.24^a^ ± 0.31	0.203	<0.001	<0.001	<0.001
Acid detergent fiber	42.42^c^ ± 0.24	43.07^cd^ ± 0.57	43.61^d^ ± 0.26	44.26^de^ ± 0.30	44.76^e^ ± 0.12	45.69^f^ ± 0.36	39.52^a^ ± 0.63	40.39^b^ ± 0.64	44.52^e^ ± 0.28	0.251	<0.001	<0.001	<0.001
NDF	53.53^a^ ± 0.28	55.09^b^ ± 0.94	57.22^c^ ± 0.88	58.57^d^ ± 0.13	59.10^de^ ± 0.24	59.94^e^ ± 0.57	53.50^a^ ± 0.41	54.30^ab^ ± 0.91	57.15^c^ ± 0.81	0.375	<0.001	<0.001	0.028
***In vitro* digestibility, %**													
DMD	37.84 ± 0.30	39.34 ± 0.48	40.84 ± 0.95	32.72 ± 0.48	34.98 ± 0.84	36.99 ± 0.82	40.17 ± 0.48	41.11 ± 0.02	42.72 ± 0.89	0.400	<0.001	<0.001	0.289
OMD	40.99^c^ ± 0.43	42.20^de^ ± 0.47	45.66^g^ ± 0.67	35.93^a ^± 0.76	36.72^a^ ± 0.68	38.85^b^ ± 0.78	41.50^cd^ ± 0.60	42.86e^f^ ± 0.33	43.47^f^ ± 0.38	0.341	<0.001	<0.001	0.004

However, this study showed a decrease in DM content compared to before fermentation, with a decrease rate of around 7.12%–7.94% (before fermentation, DM content was about 90.62%, Table 1). The decrease in DM content in this study was caused by the addition of 10–40 ml of distilled water during the inoculant-bran mixing process, which was supposed to keep the bran slightly moist to support the fermentation process. Microbes need media containing water and organic materials such as carbon, nitrogen, and other organic ions [20]. However, some of the water containers will evaporate during the fermentation process [21]. Moreover, the decrease in the DM content of fermented bran is caused by the inoculants use of several nutrients, particularly as a source of energy during the cell multiplication process. Similar conditions were reported previously [13,22], where the DM bran content decreased during the fermentation process.

The DM content of fermented bran produced in this study was slightly lower than that of fermented bran DM reported previously [12]. The DM content of fermented bran ranged from 88.5% to 88.9% for all SC yeast application treatments. Furthermore, Ahmad et al. [22] produced 89.8% DM in bran fermented using *Aspergillus flavus* for 96 h. The lower DM content of fermented bran obtained in this study may be due to differences in the DM content of the bran raw material used, type of inoculum, and duration of incubation time. The difference in the quality of the fermented products is largely determined by the different capabilities and specifications of the metabolic process of the inoculum used as a fermenter agent.

The OM content of bran due to SC and SBP inoculation treatments increased by 1.76% and 1.88%, respectively, compared to the OM content of the raw material before fermentation, which was 83.49% (Table 1). A strong interaction between increasing the dose and the type of inoculant can occur because a high population of microorganisms during the fermentation process can impact the level of OM due to the fermenter cell biomass formed. The increase in OM content in the fermentation process reflects the amount of fermenter/inoculant cell biomass [9,6].

### Crude protein content

The higher CP content in the SBP inoculation treatment was thought to be due to the higher microbial fermentation activity found in SBP during the fermentation process, which changed the compounds present in the substrate for forming cell proteins and cell population propagation. The number of microbes and nutrients in the substrate is out of balance the more active the fermentation. Microbes enter the stationary phase faster because they don’t have enough nutrients [23].

Microbes can produce enzymes, and microbes in SBP produce enzymes that can degrade complex compounds into simpler compounds and synthesize proteins for their cells, which results in an increase in bran protein. Other studies have also reported the same thing; namely, that fermentation activity can increase the CP content of fermented feed raw materials [6,9,11,23]. This happens because, during the fermentation process, there is an increase in reducing sugars and dissolved proteins due to the degradation of carbohydrate and protein components in the fermentation process. This fermentation process will lead to an increase in the process of overhauling the structure of complex OM into simpler structures. During the fermentation process, proteolytic activity breaks down protein into amino acids and increases diluted protein [21]. Therefore, it is produced from the fermentation process is a feed ingredient with a higher protein content than the basal material.

The interaction effect between the type of inoculant and the dose of inoculation showed that the two treatments influenced each other. The positive interaction effect between the type and dose of inoculants indicates that the effect of increasing the inoculant dose is influenced by the type of inoculant and *vice-versa*. It can be explained that the interaction between treatment factors occurred simultaneously, where increasing the inoculation dose linearly increased CP in all treatment interactions. The best interaction has been evaluated, resulting in the interaction of the SBP inoculant type with a 6% inoculation dose, which resulted in a CP content of 6.32%. However, the CP content in this study was much lower than that reported by the National Research Council [25] (6.32% *vs.* 12.9%) in unfermented bran.

### Crude fiber content

The inoculation that produced the lowest CF content in this study was SC compared to other types of inoculant treatment. The CF content values for each treatment can be seen in Table 2. The low content of CF in SC inoculation treatment compared to other treatments due to SC is an inoculant from a group of fungi that has the ability to produce a higher group of cellulase enzymes for breaking down lignocellulosic bonds so that the compound-complex carbohydrates, such as CF, break down into simpler carbohydrates that are more soluble. The *β-1,4-glucan* bond in cellulose will be cut by the activity of the cellulase enzyme, which belongs to the glycoside hydrolase enzyme group. This was confirmed [20,25], which stated that fungi could secrete three cellulases, namely *endo-β-1,4-glucanase, cellobiohydrolase, *and* cellobiose *or *β-glucosidase* dissolved [27].

Increasing the inoculation dose decreased the CF content of fermented bran linearly (*p* < 0.05). This is caused by the intensification of the fermentation process and substrate degradation with the increasing amount of inoculated microbial biomass. Immediately after the inoculation process was carried out, the large amount of initial biomass allowed the production of the cellulase enzyme group to also increase during the fermentation process. The resulting cellulase enzyme will then work according to the target of the enzyme on the substrate; this is commonly referred to as “*lock and key systems.” *As explained by [28], the production of cellulolytic enzymes is only stimulated in the presence of a substrate, and the enzyme works more effectively when widely accessible sugars are available. Furthermore, Bidura and Siti [29] stated that a group of cellulase enzymes, such as cellobiohydrolase, can attack the crystalline part of cellulose, and the endoglucanase enzyme can attack the amorphous structural part of cellulose. In contrast, the *β-glucosidase* enzyme will break down cellobiose into glucose.

The interaction of inoculant type and inoculation dose significantly reduced the CF of fermented bran (*p* < 0.05). It provided positive benefits, where each type of inoculant has a specific ability to degrade CF, and the activity becomes more intense as the inoculation dose increases.

### Extract ether content

The EE content of fermented bran was significantly affected by each treatment factor, albeit partially. However, their interactions did not produce a different response to the EE content of fermented bran. The overall treatment resulted in EE content ranging from 2.61% to 5.51%. The percentage of EE content of fermented bran due to the influence of SC inoculants showed a decrease of 0.28% from the percentage of the bran before fermentation, which was 3.26%. The decrease in EE content in fermented bran occurs due to the action of yeast cells (SC), which degrade complex organic materials, including fat, to meet the need for carbon substances. Saunders [30] stated that there are three main fatty acids in bran and bran, namely palmitic, oleic, and linoleic fatty acids. Crude rice bran oil contains 3%–4% wax and 4% unsaponified lipids. Perceive the trend of decreasing EE content in bran due to fermentation using SC provides a distinct advantage because it is known that bran has a fairly high EE content, which can interfere with the storage process, especially in areas with humid tropical conditions. In addition, feeding ruminants with excessive fat content will have a negative impact on fiber fermentation activity in the rumen.

The EE content of fermented bran increased concomitantly with the increase in the inoculation dose. The EE content in succession from lowest to highest was owned by SC treatment (3.58%), EM4 (4.13%), and SBP (4.30%) (*p *< 0.05). The contribution of the EE portion from the inoculant cells causes the increasing linear EE content with increasing inoculant dose. When an analysis is performed, the chemical composition is also counted as part of the EE content of fermented bran.

### Gross energy content

Gross energy (GE) is the energy contained in the feed used by livestock for maintenance and production. The GE content of fermented bran in the study ranged from 3,145 kcal GE/kg to 3,361 kcal GE/kg. Similar results have been reported [12], who noted that the GE of rice bran fermented using SC at 0.2% and 0.4% resulted in GE of 3.312 kcal GE/kg and 3,326 kcal GE/kg, respectively. However, it is lower than that reported [31] in unfermented rice bran, which is 4,500 kcal GE/kg. The difference in GE content may be due to the different sources and types of bran-producing rice used. As Mapiemfu et al. [32] stated, seasonal differences, rice variety, land planting, and processing procedures greatly affect the energy content and digestibility of rice and its by-products.

### Cellulose, hemicellulose, and lignin content

The fermentation process shows that microbial metabolic activity is cellulolytic and can degrade CF because it produces the extracellular enzymes cellulase and hemicellulase so that the CF content decreases. Microbes added during fermentation can break down more complex components into simpler compounds that are easier to digest. Fermentation by microbes will remodel the structure of the cell wall network, break the lignocellulosic bonds, and reduce lignin levels. This is in accordance with the opinion of Ranathunga et al. [33]. The effect of fermentation on CF is the breakdown of complex substances contained in the substrate by microbial enzymes, such as the breakdown of cellulose, hemicellulose, and their polymers to produce simple sugars and CF derivatives.

Such as cellulose, hemicellulose is a polysaccharide compound composed of glucose linked via *(1–4) glycoside* bonds. Some hemicellulose is known to be digestible by strong acids and bases. In plant cell walls, hemicellulose usually binds to lignin to form lignocellulose compounds [34]. Only microbes that produce cellulase enzymes can cleave the (1–4) glycoside bonds.

Lignin is a component of fiber fraction that strengthens the structure of plant stems, which makes it difficult to digest. Fermentation using SBP showed a significant decrease in the lignin content of fermented rice bran compared to the other treatments. The lignin content due to the effects of SBP, EM4, and SC inoculation, respectively, was 11.21%, 15.22%, and 16.26%.

Likewise, with the effect of the inoculation dose, the application dose of inoculants at 6% with a lignin content of 12.12% is significantly lower than the doses of inoculation treatment of 2 and 4%, which have a lignin content of 16.43% and 14.15%, respectively.

### Neutral detergent fiber and acid detergent fiber content

The ADF content fraction refers to the residue not dissolved after being boiled with a strong base and strong acid. The components of the ADF fraction include cellulose, hemicellulose, lignin, and silica. Table 2 shows that the SBP treatment significantly produced the lowest ADF content compared to other inoculant treatments. Reciprocally, the SC inoculation treatment showed that the ADF content was significantly lower than the ADF possessed by the EM4 inoculation treatment.

The low ADF fraction possessed by the SBP inoculation treatment due to microbial action contained in the SBP inoculants had a higher ability to release or separate hemicelluloses bound to lignin that compose the cell walls of fermented bran. In addition, some of the hemicelluloses can be digested, causing the content of the ADF fraction to be low. Feed ingredients with low ADF values have high-value benefits for livestock production. Pratama et al. [35] reported that SBP supplementation in swamp forage, which was high in fiber content and aged for a long time, showed a significant effect on CF digestibility *in vitro*.

The content of NDF in fermented bran was significantly influenced by the type of inoculum treatment. The EM4 treatment showed a different response to the NDF content of fermented bran, which produced the highest NDF value and showed a significant difference compared to the SC and SBP treatments, which produced lower NDF. The SC and SBP treatments themselves produced no different NDF content.

The content of the NDF fraction refers to the amount of residue from the cell components that make up plant tissue that does not dissolve after being boiled with a neutral detergent. The dissolved compounds are generally in the form of simple compounds contained in the cell’s contents, including simple sugars, proteins, and amino acids. At the same time, the insoluble residue consists of cellulose, hemicellulose, lignin, and silica.

### In vitro dry matter and organic matter digestibility

The level of feed degradation can be used as an indicator of feed quality. The higher the DMD and OMD of a feed, the greater the availability of nutrients that can be used to meet the nutritional needs of livestock. The purpose of determining digestibility is to get a initial estimate of the value of feed ingredients because only digestible feeds can be absorbed.

The high OMD in the 6% treatment was closely related to the DMD value in the treatment. There is a strong correlation between DMD and OMD, in that a high DMD can certainly result in a high OMD. Fariani et al. [36] stated that the breakdown of OM and DM was closely linked because most DM was comprised of OM.

The digestibility of a feed reflects the high and low value of the feed ingredients benefits. If the digestibility is low, the benefits value is low, and vice-versa. When the digestibility is high, the benefit value is also high. Fermentation efforts will be useful if the digestibility value is known. Ali et al. [37] and Lai et al. [38] stated that to achieve optimum rumen microbial growth, a balance between energy availability and NH3 in the rumen is required.

## Conclusion

This study showed that the inclusion of SBP inoculants at a dose of 6% in fermented bran was very effective in increasing and improving the chemical composition of the bran. Overall, there was a synergistic interaction between the type and dose of inoculant in improving the chemical composition and increasing the digestibility of bran in the rumen. Another *in vivo* study should look at the direct effects of different types and doses of inoculants on animals, especially how they work as potential probiotics.

## List of Abbreviations

ADF = Acid detergent fiber; CF = Crude fiber; CP = Crude protein; DM = Dry matter; DMD = Dry matter digestibility; EE = Extract ether; EM4 = *Effective microorganism-4*; NDF = Neutral detergent fiber; OM = Organic matter; OMD = Organic matter digestibility; SBP = Saus burger pakan; SC = *Saccharomyces cerevisiae*.
